# “You have to be okay with okay”: experiences of flourishing among university students transitioning directly from high school

**DOI:** 10.1080/17482631.2020.1834259

**Published:** 2020-10-26

**Authors:** Christina Volstad, Jean Hughes, Sonya L. Jakubec, Sonya Flessati, Lois Jackson, Ruth Martin-Misener

**Affiliations:** a Mental Health Nurse Clinician, Alberta Health Services, Calgary, Canada; b School of Nursing; Research Scientist at IWK Health Centre; Senior Researcher with Healthy Populations Institute, Dalhousie University, Halifax, Canada; c Faculty of Health, Community and Education School of Nursing, Mount Royal University, Calgary, Canada; d Registered Psychologist, Mount Royal University, Calgary, Canada; e Health Promotion, School of Health and Human Performance, Faculty of Health, Dalhousie University, Halifax, Canada; f School of Nursing, Dalhousie University, Halifax, Canada

**Keywords:** Flourishing, university, students, transition, qualitative, Gadamer’s philosophical hermeneutics

## Abstract

Mental health is central to overall wellbeing and, for students attending university, mental health is critical for learning and academic success. A wealth of research has focused on young people who experience psychosocial declines during academic and developmental transitions, but little is known about how young people flourish in this transition. The first to explore the experiences of flourishing among first-year Canadian university students making the transition directly from high school, this study sought to develop an understanding of: 1) *the factors that promote flourishing amidst this academic and developmental transition*, and 2) *how first-year students define and experience flourishing*. An interpretive phenomenological approach underpinned by Gadamerian hermeneutic philosophy was used to explore experiences of flourishing, using semi-structured interviews, in a sample of nine full-time, first-year university students, ages 18–20 years. What it meant to flourish amidst this developmental and academic transition and how participants defined flourishing offer new understandings of the concept associated with: *1) personal/individual aspects of flourishing, 2) contextual nature of flourishing, 3) temporality of flourishing, 4) dialectic aspects of flourishing*. Implications for practice, policy, and research in light of these new understandings are discussed.

## Introduction

Mental health is central to overall well-being, linked to better physical health (World Health Organization, 2014), and for students attending post-secondary institutions, mental health is important for learning and academic success (Schroeder & West, 2019). Individuals between the ages of 15 and 24 years are more likely to experience mental illness and/or substance use disorders than any other age group (Centre for Addiction and Mental Health, 2020), and this is a time when many young people graduate from high school and go on to pursue post-secondary education. Since the 1970s there has been a rise in the number of young people attending post-secondary institutions (Clark, 2007; MacKean, 2011) and mental illness is one of the most significant challenges faced within this context today. A wealth of research has focused on young people who experience psychosocial declines (Hunt & Eisenberg, 2010; Oswalt et al., 2020; Storrie et al., 2010); however, little is known about how young people (age 18–20) are adapting and coping well while making this academic and developmental transition.

Flourishing refers to the experience of feeling good—high levels of emotional, psychological and social functioning most of the time. As a relatively new concept, agreement has yet to be reached among scholars on how flourishing and its constituent indicators are defined (Agenor et al., 2017; Hone et al., 2014). To date, four conceptual frameworks with assessment scales have been developed for measuring flourishing: 1) Keyes’ Dimensions of Flourishing Keyes, Huppert and So’s Flourishing Scale (Huppert & So, 2013), 3) Diener et al.’s Flourishing Scale (Diener et al., 2010) and, 4) The PERMA-Profiler (Butler & Kern, 2016). These frameworks and measures have helped to define and understand the concept from a quantitative perspective but the qualitative experience of flourishing has also been minimally explored (Ashfield et al., 2012; Gokcen et al., 2012). There are even fewer studies exploring the experiences of flourishing among students during the transition to university (Knoesen & Naudé, 2017). This phenomenological study is an exploration of the experiences of flourishing among university students transitioning directly from high school to university and provides a beginning understanding of how first-year students define flourishing and the factors that promote it amidst this dual transition.

## Background

Mental health issues among post-secondary students have increased in chronicity and have involved a range of problems which can negatively affect students’ academic success (American College Health Association, 2019; MacKean, 2011). With a rise in the psychological distress of students, there is a growing awareness among post-secondary administrators of the need for more sustainable and long-term strategies to promote mental health (University of Calgary, 2020; University of Toronto, 2018). Despite recent mental health promotion efforts, strategies to improve student mental health continue to emphasize interventions to address problems and challenges, with fewer policies and resources devoted to understanding the factors that keep students well (De Somma et al., 2017).

The problem-solving orientation is similarly emphasized in the 2019 National College Health Assessment (NCHA), a survey of Canadian post-secondary students that explored their health behaviours, attitudes, and experiences over the previous 12 months (American College Health Association, 2019). The survey involved 58 campuses across Canada and 55,284 students (American College Health Association, 2019). Anxiety and depression topped the list of mental health conditions diagnosed or treated by a professional in the previous 12 months. While the survey findings reported 44.4% of students as flourishing (American College Health Association, 2019), it failed to provide an understanding of the pathways used to maintain positive mental health, the contextual factors that promote students’ flourishing, or how mental health could be protected or promoted.

### The post-secondary mental health context in Canada

Canada has one of the world’s highest quality and advanced higher education systems consisting of universities, community colleges and polytechnic institutions (Usher, 2019). In 2019, Canada implemented initiatives within several post-secondary institutions to include: 24/7 mental health supports (via phone or electronic device), expanded on-campus counselling services, new campus community spaces devoted to mental wellness, artificial intelligence-powered mental health assistants, along with supporting increases in student service fees so that more mental health services could be made available. The province of Alberta alone awarded 22 USD million to 26 post-secondary institutions to support new and existing mental health and addictions supports (Academica Group, 2020).

Programs across the country have been developed and implemented in recent years. One program based on Seligman and colleagues’ operational definition of flourishing was offered to new students at Ryerson University in Toronto, Ontario and aimed to support students while increasing awareness about the link between academic success and healthy lifestyle (Ryerson University, 2020). In the fall of 2017, seven Canadian universities partnered with the Mental Health Commission of Canada (MHCC) to pilot “The Inquiring Mind” program. This program, examines topics of stigma, coping and managing stress, and aims to empower students to understand and manage their mental health (Mental Health Commission of Canada, 2020).

Encouragingly, models using a whole school approach, such as the Comprehensive School Health Framework (Pan-Canadian Joint Consortium for School Health, 2017) to guide Mental Health Promotion (MHP) efforts at the elementary and secondary school levels are now being introduced in post-secondary settings. These mental well-being promotion efforts draw from a broad range of upstream approaches and have been incorporated into the following frameworks: The Campus Population Health Promotion Model (Patterson & Kline), Ecological Model (American College Health Association, 2018), Healthy Minds/Healthy Campuses (Canadian Mental Health Association, 2020), and the UK Healthy Universities Model (Healthy Universities, 2020). The Okanagan Charter, an international charter for promoting the health of post-secondary students, considers the social, cultural, political and economic contexts in which individuals, communities and societies are immersed and which influence individuals’ mental health (Holt & Warne, 2007).

### Developmental and academic transition

Transitions across the lifespan involve the experiences of dislocation, disorientation, and disruption, a process of adaptation and characterized as multidimensional, complex, and uniquely experienced by individuals (Meleis et al., 2000; Schlossberg, 1981; Schumacher & Meleis, 1994). The developmental and academic transitions of young people (ages 18–20) moving directly from high school to university require a rapid change and adaptation within the new post-secondary education context. Beyond the context, the period of late adolescence to young adulthood (approximately ages 18–20), involves a developmental transition in which physical, cognitive, social, emotional, and moral changes occur. Coinciding with these changes is risk-taking and experimentation with substances, sexual partners and societal rules and norms (Kloep et al., 2015). The transition involves identity exploration, self-regulation, the development of new relationships and the separation of the individual from the family (Humphrey, 2009).

The transition directly from high school to university is a highly anticipated life phase and it has the ability to affect later life adjustment (Bland et al., 2012; Pratt et al., 2000). The challenges faced in this transition include leaving the parental home, experiencing financial strain, fitting into a new environment, establishing new social networks and supports, navigating the new post-secondary environment and adjusting to higher academic expectations and commitments (Clark, 2007; Johnson et al., 2010).The inability to cope with stress during this transition can result in adverse health behaviours and can affect the mental health status of young people (Bland et al., 2012), both of which may place students at risk for withdrawing from university (Alarcon & Edwards, 2013). Some students are challenged by this transition, but others are successful at adapting and flourishing during this transition. Predictors of student success include the practice of good health habits and risk management skills, as well as positive psychological, emotional, intellectual, and social development (Park et al., 2011).

In making the academic transition from high school to university, students are expected to become independent learners, manage time more effectively, and make responsible decisions. Unlike developmental transitions, the academic transition to university is an individual choice requiring students to adapt and cope with a new environment. The cohort of Canadian students (age 18–20) experiencing this academic transition is not, however, homogeneous. Research suggests that diversity factors reflecting ethnicity, gender, sexuality, disability, culture, and international, and socio-economic status may significantly heighten stress and psychological difficulties during this dual transition period (Michalski et al., 2017; Usher, 2019). Despite the challenges and developmental tasks, or perhaps because of these conditions, flourishing, or the experience of feeling good (high levels of emotional wellbeing or hedonic wellbeing) and functioning well (high levels of psychosocial wellbeing or eudaimonic wellbeing) most of the time (Hone et al., 2014), is possible for this population.

### Flourishing

Flourishing is a relatively new concept and agreement has yet to be reached among scholars on how flourishing is defined, or its constituent indicators (Agenor et al., 2017; Hone et al., 2014). To date four operational definitions of flourishing with assessment scales have been developed for measuring flourishing: 1) Keyes’ Dimensions of Flourishing Keyes Huppert and So’s Flourishing Scale (Huppert & So, 2013), 3) Diener et al.’s Flourishing Scale (Diener et al., 2010); and 4) The PERMA-Profiler (Butler & Kern, 2016). Despite the lack of consensus on a theory, conceptualization and definition of flourishing, there is strong agreement among all operational definitions, that flourishing is a combination of a set of hedonic and eudaimonic indicators, with overlap in the areas of: 1) positive relationships, 2) positive affect (interested) and engagement, and 3) meaning and purpose (Hone et al., 2014).

Although there are slight differences in how these indicators are defined, the definitions generally come from the same body of literature on well-being. Keyes’s model is the most comprehensive in the area of social well-being (Hone et al., 2013) and uniquely situates flourishing within a model of mental health (Dual Continuum Model of Mental Health) (Keyes). There are four main theories proposing similarities and differences among the constituent indicators of flourishing; however, a lack of consensus exists on a single measure to examine the prevalence of flourishing among populations and cross-culturally. The four theorists agree on similar indicators, but at times different language is used to describe these indicators (e.g., purpose in life vs. purpose and meaning). In light of these contributions and what others might view as an omission of certain indicators (such as virtue, physical health, religion, security, grit and growth mindset) no one particular operational definition was excluded from framing this study. Instead, the value of having an awareness of all indicators and the potential outliers of flourishing shaped the lead researcher’s pre-understandings and provided a space to remain open to all the possibilities of how university students define and experience flourishing while transitioning directly from high school.

The frameworks and measures have helped to define and understand the concept from a quantitative perspective but the experience of flourishing has been minimally explored from a qualitative perspective (Ashfield et al., 2012; Gokcen et al., 2012). Studies exploring the experiences of flourishing among students living through the transition from high school to university are even fewer (Knoesen & Naudé, 2017). Predictors of flourishing within the post-secondary context include academic achievement (Antaramian, 2015; Howell, 2009), socially supportive environments, ease with transitioning, sense of belonging, civic engagement (Fink, 2014; Nicotera et al., 2015) and study engagement and involvement (Bowman et al., 2010; Low, 2011). To the authors’ knowledge there are currently no studies specifically exploring the experiences of flourishing among university students transitioning directly from high school within the Canadian context.

## Methods

### Setting

The site of this study was a university located in a Western Canadian city of over a million people. During the 2016/17 academic year, the full-time student population at this undergraduate university was approximately 10,000. International students comprised 3.5% and Indigenous students 5% of the total population across all programs. Residence complexes accommodated up to 1,000 students. With 12 degree programs, and 32 majors, the average class size was 29 students in the 2016/17 academic year. The campus had competitive sports teams and a number of other activities such as student and peer support groups, volunteer and employment opportunities, recreational programs/intramural/drop-in sports, workshops, and international committees. In 2017, the university became one of seven Canadian post-secondary institutions to partner with the Mental Health Commission of Canada to pilot “The Inquiring Mind” project referred to above, which was implemented to train first-year students on how to better understand and manage their mental health with a focus on stigma reduction and building resiliency (Mental Health Commission of Canada, 2020).

### Population

The target study population was students between the ages of 18–20 years who transferred directly from high school into their first year of university and enrolled in a full-time degree program. This cohort was chosen due to heightened challenges of first year that students may experience, including moving away from their parental home, financial strain, adjusting to higher academic expectations, navigating a new social environment and establishing new social networks and supports (Clark, 2007; Johnson et al., 2010).

### Sample

Interpretive phenomenological approaches tend to produce a large amount of data; therefore, smaller sample sizes are recommended (Smith & Osborn, 2015). Given the lack of any precise guidelines for hermeneutic inquiry (Moules et al., 2015), the guiding principle for determining the number of participants for qualitative studies is data saturation (Mason, 2010). For this study data saturation was reached when further interviews failed to provide a clearer understanding of the experience (Laverty, 2003) and there was diversity among participants in terms of gender, ethnicity, degree program and living arrangements.

Predictors of flourishing within post-secondary contexts include student engagement, involvement and academic preparedness. Since students were not given a rating scale to pre-determine flourishing, students who self-identified as flourishing (feeling good and functioning well most of the time) while making the transition directly from high school to their first year of university were eligible for inclusion. Participants were included based on the following criteria:

- Age 18–20
- First year of university
- Graduated from high school in the last 12–18 months
- Enrolled in a full-time degree program
- Perceived self as feeling good and functioning well most of the time

### Data collection

Forty recruitment posters were displayed in designated pre-approved common areas on campus. The posters used plain language, various fonts and colour to draw students’ attention to the study. Recruitment efforts involved contacting and meeting with various stakeholders engaged with first-year students. Faculty chairs and student advisors agreed to forward emails to 1st-year students across all degree programs, and post or share recruitment posters and emails on approved campus social media outlets. According to the Keyes’ Dual Continuum Model of Mental Health, it is possible for individuals to flourish with a mental illness; therefore, students were not excluded from the study for voluntarily disclosing a diagnosis of mental illness as long as they met the inclusion criteria (Keyes).

Participation in the study required written informed consent. Interested students who met the inclusion criteria were given an overview of the study, a copy of the consent form and encouragement to contact the lead researcher with questions. In-depth semi-structured interviews were the prime method for collecting data in this study (Creswell, 2013; Smith et al., 2009). The interview took a conversational nature to become fully immersed in the phenomenon of interest (Cohen et al., 2000; Fleming et al., 2003) and stay as close as possible to the lived experience (Laverty, 2003), through “careful and mindful listening and skilled questioning …” (Moules et al., 2015, p. 89). Interviews varied in duration but overall were between 60 and 120 minutes. The semi-structured interview guide was informed by the operational definitions of flourishing and indicators found within each of the definitions (Butler & Kern, 2016; Diener et al., 2010; Huppert & So, 2013). Interviews were transcribed by the lead researcher verbatim and pseudonyms were used to protect anonymity.

### Trustworthiness

The goal of hermeneutics is to provide the reader with proof that what is presented is credible, believable and recognizable (Moules et al., 2015). Establishing trustworthiness involves four key concepts common to all qualitative methodological approaches: credibility, transferability, dependability, confirmability (Creswell, 2013; Lincoln & Guba, 1985). Considering *credibility*, participants were chosen for their ability to shed light and bring knowledge to the topic and expand understanding of the phenomenon of central focus (Moules et al., 2015); for this study, flourishing.

Understanding the university context involved the lead author’s consultations with a few faculty and a health promotion specialist working directly with students and reviewing select university reports and the university website to understand student mental health programs and services offered on campus. Prior to engaging in semi-structured interviews, participants were asked to provide some socio-demographic information. A reflective journal was maintained throughout the study. Reflections were discussed with the co-authors and linked to theory as a way of ensuring credibility.

The issues of *transferability* are considered, however in hermeneutic inquiry generalizability and transferability are not where truth lies; instead, truth lies in the extent to which the research raises more questions and extends understanding (Moules et al., 2015). No two universities are alike. Their different demographic factors and use of different approaches to promoting student mental health, and models or frameworks constitute unique contexts which become pivotal to understanding the transferability of interview findings.

*Dependability* of the data and results was maintained through reflective journal and field notes. Initial notes were taken immediately after the interview. Brief phrases and words were written as a reminder of critical points in the conversation—further expansion and reflection followed. Each interview was transcribed by the lead author, indicating pauses, mishearings, mistakes and speech dynamics (Biggerstaff & Thompson, 2008). Reflective notes were written after each encounter with the data and after weekly discussions with the second author. Reviewing the literature facilitated further reflective understanding.

The shortcomings of the study methods and potential effects and a detailed in-depth methodological description to allow for scrutiny of the research were provisions made to ensure *confirmability* of the data collected (Creswell, 2013; Moules et al., 2015; Shenton, 2004).

### Data analysis

Phenomenology contains two main approaches: descriptive and interpretive. The philosophical underpinnings for this study are rooted in interpretive phenomenology, an approach consistent with the nature of the research question. Within the interpretive phenomenological branch, Gadamer’s hermeneutic phenomenology provided the philosophical framework for this study. Hermeneutics recognizes that a phenomenon cannot exist without context and the awareness of preunderstandings, central to understanding. The following Guidelines for a hermeneutic practice, underpinned by Gadamer’s philosophy, guided the hermeneutic inquiry:

1. The way of hermeneutic practice is determined by the phenomenon, not the method.

2. Hermeneutic practice requires a disciplined (phenomenological) focus on the particular.

*3. Hermeneutic practice requires that we be vigilant and open in our encounters with the life world*.

4. Reading in the hermeneutic tradition involves a practice of learning to read self and world differently.

5. The nature of hermeneutic practice is dialogical.

Hermeneutic analysis is a process of co-construction between the researcher and participant, engaging in a hermeneutic circle—an expanding circle of ideas that is created by using a back and forth process with study participants, which helps the researcher to discover true meaning in the experiences (H.G. Gadamer, 2013, p. 302; Wojnar & Swanson, 2007). Central to hermeneutic analysis is the notion that neither the whole, or the individual parts, can be viewed separately and must be understood in reference to one another (Laverty, 2003; Smith et al., 2009) and multiple realities and pre-understandings are brought to light amidst a dialectical movement among the parts and the whole. This interpretation arises from this fusion of horizons of both participant and researcher (Laverty, 2003). Interpretive phenomenological analysis is strongly influenced by Gadamer’s philosophical underpinnings; therefore, the following step by step approach outlined by Smith et al. (2009, pp. 82–103) was used to guide the analysis phase:

1. Reading and re-reading

2. Initial noting

3. Developing emergent themes

4. Searching for connections across emergent themes

5. Moving to the next case

*6. Looking for patterns across cases*

Taken together, this process enabled the formation of a deeper understanding of flourishing for the first year students characterized by four interrelated emergent understandings: *1) personal/individual aspects of flourishing, 2) contextual nature of flourishing, 3) temporality of flourishing, and, 4) dialectic aspects of flourishing*. These understandings are the central focus of this paper.

## Findings

### Demographics

Nine full-time students volunteered for the study identifying as female (n = 8) and male (n = 1) and were registered in the following programs: Bachelor of Nursing (n = 6), Bachelor of Education (n = 1), Bachelor of Business Administration (n = 1), and Bachelor of Child Studies (n = 1). Participants’ diverse ethnic backgrounds included: Caucasian, Caucasian/Metis, Caucasian/Filipino, Chinese/Irish/English/Scottish, Vietnamese, Chinese, Russian and Pakistani. Two participants lived in campus residence, four were living with parents or extended family, and three lived off-campus independently in a family-owned apartment, or with extended family. All students were raised in Western Canada including two who had immigrated before the age of 10.

### Emergent understandings

#### Personal/individual aspects of flourishing

University provided a context in which participants could enhance and improve areas of personal strengths and personal challenge. Participants shared personal coping and self-care strategies (exercise, healthy diet, good sleeping habits) as part of a regime that promoted flourishing in first year. Most participants described personal characteristics such as a willingness to adapt and step outside their comfort zone that promoted flourishing amidst the transition. Taking advantage of, and being flexible and open to, opportunities for becoming involved on campus contributed to making social connections and feeling a sense of belonging in the university environment. Mimi explained the adaptive quality in this way:

… I would say to flourish in the first year would be, to be able to adapt to any changes. To be resilient in the changes and to be able to put yourself out there because to be closed up in the first year of university it’s really hard to make the connections and it’s hard to feel like you belong unless you open yourself up and put yourself out there … (Mimi, Int. 4)

For most participants, asking questions and asking for help were personal challenges to be overcome in first year in order to take advantage of the many on-campus supports, and resources to promote wellbeing, academic success and expand social networks. As Alex explained:

… just knowing when to ask for help … because before in high school … it’s not like you couldn’t ask, it’s just that there was this stigma … What are you saying? That’s a dumb question? … but I feel like it’s important to ask the questions because that’s like what you’re going to need to go on. First semester I definitely didn’t do that, no confidence at all. I think it was because it was a new place and not that many friends … (Alex, Int. 9)

Some participants indicated they experience physical and mental health challenges during the transition. However, many positive insights and new personal understandings arose from this place of challenge in terms of learning better coping and self-care strategies. In the pre-transition period, Penny developed a fear of being vulnerable which followed her to university, making it difficult to trust others and affecting her ability to establish social connections. This was further complicated by the exacerbation of symptoms of her pre-existing health challenges and feelings of being invisible and judged by others. Penny understood flourishing to be routed in her personal journey of finding meaning and coming to terms with having chronic health conditions. As she commented, *“… you have to settle with feeling ok and functioning ok …”*. The ups and downs linked to her personal health challenges made the process of flourishing in first year difficult but during the challenging times there was no doubt that Penny felt a sense of accomplishment for her academic success because as she noted,

… I was feeling okay and functioning okay … I don’t think there’s ever going to be a good … it’s a chronic condition … I would say that I was functioning ok which is pretty awesome. After a while I found my groove. I found a routine that worked kinda well … So I was feeling like in the moment I was learning all of this new stuff … (Penny, Int. 7).

What it meant to flourish when making the transition to university was less about feeling good and more about the functioning well components of flourishing. In this way, flourishing depends on the movement of a person, in relationship and context. The essence of all participants’ experiences of flourishing was, in the end, more about the *personal* growth than the *academic* growth that had occurred as a result of pushing through and growing in challenging times.

For Carlos, being happy was not at the heart of flourishing nor was it a precursor to functioning well during this transition. Happiness was characterized as a result of functioning well and stepping outside of one’s comfort zone and growing as a person. Carlos stated that:

… flourishing would be stepping back, taking account … acknowledging that you’re scared. That university itself is terrifying to a high schooler … after you’ve acknowledged you are scared, then next you step outside of that boundary. Because now you know where the line is, you know what you’re afraid of and then if you explore something that you’re not used to then you can grow as a person … (Carlos, Int. 8).

Josephine shared her thoughts and understanding of the need to push past her fear of emotional vulnerability in relationships. This is a goal that she would like to work on even more in the future which emphasizes that time passing and the steps towards goals are symbolic of continued personal growth. As Josephine shared, putting herself more emotionally into relationships and allowing herself to be vulnerable, encouraged more meaningful relationships that lead to an expansion of her social support networks. Observing and experiencing her friend’s ability to be vulnerable allowed her to consider what it might be like for her to be emotionally vulnerable with others. She commented that:

… I would really like to see myself put myself more emotionally into relationships and like friendships. I find it really easy to be vulnerable with my family members … but people who are not in that little circle I often do not tell anything that’s going on with me personally … but I’ve had a way richer experience because my friends have done that to me. I’ve been able to help them and I felt super accomplished because of that and I felt like I’ve grown closer in my relationships with them and I kind of haven’t done that in return so that’s a goal that I have. Just being a little bit more vulnerable … (Josephine, Int. 5).

Emerging from these challenges depended on the individuals’ strengths and supports that could be utilized to help them overcome their challenges and experience personal growth. Of significant importance was the perception of the challenge(s) and the interpretation of adversities that determined whether they would flourish, and this is consistent with what is already known (as cited in Yeager & Dweck, 2012).

#### Contextual nature of flourishing

Flourishing within this transition was clearly an active process involving participants shaping and interacting with family, their living environment, and their community. Participants shared the many accounts of how context contributed to what flourishing meant to them and to the positive and challenging aspects of the context in which they were immersed.

### The family context

All participants described family/parental support and challenges as a significant element of flourishing in first year. Not only did family support for monetary needs (financed accommodations, tuition and food) significantly reduce stress in first year, but it also provided stable and dependable emotional support. As Gabby describes,

… my parents because they had supported me through so much without them I don’t think I would be able to make all of these transitions by myself. Just having them there to provide me with physical support and economic support and anything that I could ever need. Even though I do fight with my parents … (Gabby, Int. 6).

In the pre-transition period, challenging times brought family members together to support each other amidst the transition to university as the following quote from Pam indicates.

… one of my older brothers passed away last year, he was living with [my older sister] … when he passed she was living alone and I just couldn’t not allow myself to not live with her especially when I had that option because she just lost one of her best friends as did I … we needed each other … (Pam, Int. 3).

Family cultural practices (e.g., fasting) shared in the pre-transition and transition period also contributed to their ability to flourish, *“ … currently I’m fasting so that’s definitely a wellbeing kind of thing cause not only is it good for you—like mentally … our values and our beliefs … my parents have instilled that in me … ”* (Alex, Int. 9)

### The living environment

Prior to entering first year, participants made intentional and thoughtful decisions about where they wanted to live in terms of how it would contribute to their academic success and wellbeing in first year. The living environment was experienced by all participants as having some positive and challenging aspects contributing to their ability to flourish. Some participants’ living arrangements had not changed from their pre-transition living environments which facilitated receiving continued support from parents and siblings, but it conflicted with their developmental needs of becoming more independent.

Experiences living away from the parental home provided opportunities to develop life skills, establish social support networks, explore identity and build confidence as Rose explains.

… I’ve definitely grown up because I’m living on my own and I’m responsible for feeding myself and going to the dentist … it’s really made me my own person and I realized that I don’t have to rely on my Dad for everything … you know and I can figure things out for myself … so I’ve learned how to be less uptight and anxious about things … (Rose, Int. 1)

### The university environment

All participants reported that the small class sizes and small student and faculty population at the university made a positive difference in feeling a sense of belonging to the university and contributed to their establishing social networks and getting to know faculty.

… It really helps here at [the university] because the class sizes are really small compared to [other universities] where it’s 200-300 people. Whereas here it’s 35 so you really get to know your professor and they know you by name and give you really good feedback on your work and make really good personal connections with you … (Rose, Int. 1)

Several students shared that high school had not prepared them for the higher expectations in university. *“ … high school didn’t really prepare me for the writing … I also found the course load and specifically in bio in high school we would spend around 2–3 weeks per chapter but in uni., we’re now doing pretty much a chapter a week …* ” (Charlotte, Int. 2)

Faculty who were approachable, and willing to help, made students feel like they were more than just a number and promoted flourishing.

… They’ve all been really good so far … they all um focus on the student as a whole instead of like another number … it’s nice to know that they actually know your name and if you need any help or anything you can just come in and talk to them, it’s not intimidating or anything like that … (Gabby, Int. 6)

Informal and formal programs, for example, the Peer Learning Program, created opportunities for on campus involvement and a safe environment to receive support. Penny explains that she loved peer learning and that she felt comfortable asking questions in this setting.

… … I knew that it was their job to understand more things than I did and it was wonderful to not have to be the smartest person in the room … there was definitely things that I didn’t know that I needed to ask clarifying questions for, that my fellow learners didn’t judge me for and that the tutor didn’t judge me for either … (Penny, Int. 7)

Participants shared that working with peers they didn’t know—or who had a different work ethic, or personality traits—was at times uncomfortable and challenging. However, this became an opportunity for personal growth as Carlos explains.

… for one of my classes in first semester … I was placed into a group of three extroverted rap fans … the stereotype wouldn’t have meant top quality grades … but I left with an A+ and a lot of this was due to their input and the way that they worked together. So working in groups and seeing all these personalities disproves some of the stereotypes of what makes someone effective … you can’t just assume that because someone’s extroverted, peppy or talks as if they know what they’re doing … (Carlos, Int. 8).

Compared to high school, a more intense course load made it easy to lose touch with off-campus friends and finding that balance between school and socializing was challenging, took time, and for many, this did not occur until second semester. Charlotte explains this as follows:

… In my first semester I was kind of sticking with the people that I know because you know … transitioning … you don’t really know what to really expect, you kind of hang on to people who you are familiar with. Then in the second semester I found myself kind of branching out and making new friends … it was a pretty good experience … (Charlotte, Int.2).

### Community environment

Off-campus involvement was experienced by many participants as contributing to their ability to flourish in first year. Some participants described off-campus friendships in terms of being more meaningful and providing them with the much needed break from more academically focused social networks on campus. Mimi explains her experience with off-campus friends in the following terms:

… Whenever I’m with them (off campus friends) I’m like, we never talk about homework because we’re all in different faculties and we’re all in different schools. So whenever we hang out or anything it would be like just to relax, like never on homework it’s more on how our lives are going, we talk about how our wellbeing is going basically like … we plan more fun things like ice cream or like movies, anything like that … (Mimi, Int. 4).

For some participants, the off-campus contexts (attending church, volunteering, paid employment and socializing with off-campus friends) promoted flourishing, thus highlighting the multitude of contexts and many ways in which first-year students can flourish. Josephine describes her personal experience with attending church.

… another one that for me … encompasses all aspects of health is going to church … So it’s like this group of people that just wants to feed themselves spiritually and just take a break once a week. Sometimes we’ll have meals together after church. At first everyone was just kind of strangers and now it’s just kind of my friends away from school and it’s been awesome it’s been refreshing … I think it’s really uplifted me … (Josephine, Int. 5)

#### Temporality of flourishing

The life experiences leading up to the transition to university, flourishing amidst the transition, and participants’ sharing goals and future aspirations speaks to the temporal nature of flourishing and the realization that we are always in flux with our history (McManus Holroyd, 2007). In many ways, flourishing was similarly experienced by participants as a *process* rather than a destination or *endpoint* as expressed by Josephine.

… at the start of semester, I wasn’t involved in anything school-related like except for academics … I kind of closed myself off a little bit. I was in the library studying 24/7 or I was at the gym and I maybe had like 1 or 2 friends. But in the start of the next semester, I made a goal for myself, to kind of like branch out a bit more … that’s been a huge shift from my first semester where I was more on the grind, I was still doing good, I was still pretty healthy but my health had improved significantly since I branched out … (Josephine, Int. 5).

Development takes time, as several participants expressed, not yet feeling like an adult and in essence characterizing the road to adulthood as temporal. Rose indicates that she does not yet feel like an adult.*“ … I don’t have a job … My dad is loaning me the money to go to university … I think maturity wise I’ve grown up a lot … I still do stupid stuff sometimes because I’m still a teenager, you know coming to [name of province] the legal drinking age is 18 … so sometimes I let myself go a little too far with that but that again is just a learning experience … ” (Rose, Int. 1).*

Likewise, the temporal nature of flourishing was influenced by participants’ life events leading up to university and contributed to their experience of flourishing in first year. Gabby illustrated this experience with immigrating to Canada and the challenges her family had building a new life. These experiences have contributed to her grit and perseverance in pushing through tough times in university. This is further illustrated in the final emergent understanding whereby challenge is a necessary component of Gabby’s experience of flourishing.

… my family has gone through so much stuff going through all the changes and there has been so much stuff during my childhood … [my dad] used to work in [name of city} so when he’d be there he would be fine, but he would come home he would end up drinking a lot … so that was a lot of my childhood and just being able to kind of push through that … I think that factor kind of helped me build the emotional resilience and kind of stability … (Gabby, Int. 6)

#### Dialectic aspects of flourishing

Participants experienced many personal and contextual challenges that influenced fluctuations in their emotional wellbeing and challenged their ability to flourish in first year. Challenge was experienced by all participants and interpreted to be a necessary ingredient of flourishing within this context. Carlos shared his thoughts on how his doubtful and quiet nature helped him flourish within the university context.

… I doubt a lot of things and that’s a good strength so then I can go back and question it and work at it. Another strength … I’m so quiet, I guess then it means I can listen more … I don’t have to prove anything because I’m more interested in what you can teach me than what I can say about what you taught me … (Carlos, Int. 8)

Mental health struggles were perceived as a way of developing positive coping strategies for Josephine that helped her flourish in her first year.

… I think that the struggles that I’ve been through with my mental health … have kind of formed these coping mechanisms which have turned into very healthy coping mechanisms, which is something that I’m very fortunate … (Josephine, Int. 4)

## Discussion

This study found that among students transitioning to university who perceived themselves to be functioning well, the essence of the experience of flourishing involved “challenge” as a universally experienced characteristic. The research indicates that flourishing is not simply a compilation of a set number of positive indicators at a particular point in time but that challenge is critical to flourishing, because challenge and flourishing are connected and interdependent as suggested by Lomas and Ivtzan (2016) in their discussion of the components of a dialectical process.

Personal strengths and areas of personal challenge were important aspects of the experience of flourishing for participants who encompassed a mindset in which challenges were perceived from a place of possibility and opportunity, and which arose from having the courage to be vulnerable. Participants’ descriptions of the challenges included: facing fears about moving into residence, having the courage to persevere in the face of academic challenges, the courage to expand horizons and take advantage of opportunities within the university environment, the courage to continue with one’s studies after a painful family loss, and challenges with personal chronic health issues.

The level of perseverance, determination and passion for achieving long-term goals was a key characteristic among all participants in this study. Grit is a personality trait defined simply as “perseverance and passion for long term goals” (Duckworth et al., 2007, p. 1087), and although grit is absent from the four operational definitions of flourishing it was present in the participants’ interviews. In addition, while a growth mindset also was not an indicator included in any of the four operational definitions, it appeared in the interpretation of the meaning of participants’ experiences of flourishing. A growth mindset is defined in terms of embracing challenges, persisting in the face of setbacks, viewing effort as the road towards mastery, and learning from criticism as well as from the success of others (Dweck & Leggett, 1988; Mindsetworks, 2017). All participants shared experiences that reflected having a growth mindset rather than a fixed mindset which is one that is static, views effort as fruitless, ignores feedback and feels threatened by the success of others (Dweck & Leggett, 1988; Mindsetworks, 2017).

Transitions are processes that take place over time; involve development, flow, or movement from one state to another; include stages or phases; and result in changes in identity, roles, relationships, abilities, and patterns of behaviours (Schumacher & Meleis, 1994). In our study, flourishing was similarly experienced as *a process* that took place over time in terms of the personal growth that participants experienced and the time it took to arrive at a place where they could perceive themselves as flourishing in their first year. Flourishing was a *process*, not an *endpoint*. Development takes time, and as several participants expressed, they were not yet feeling like an adult. In essence, they characterized the road to adulthood as temporal, and likewise flourishing was characterized as temporal and as affected by participants’ personal histories. Framed by development and movement over time, flourishing for first-year university students was expressed through an ecological frame of personal, relational, dialectic, contextual, and temporal factors. Although there are no absolute truths in understanding as understanding is never final, we can say with confidence that flourishing is not a “primarily private phenomenon” (Keyes, p. 121). Flourishing can only begin to be understood in terms of its relationship with context, or perhaps more accurately a myriad of contexts (family, living environment, university and community). Participants shared many accounts of how context contributed to what flourishing meant to them and to the positive and challenging aspects of the context in which they were immersed. In terms of the developmental transition that participants between the ages of 18–20 experienced, university is a context that encourages and influences the movement from adolescence to young adulthood. The university context required that the student s be active in the process of taking advantage of opportunities in their environment to build confidence and skills that would help in the subsequent years of their study and in life. The personal strengths, challenging experiences, and opportunities leading up to university influenced how participants flourished during the transition which demonstrates that historical awareness is always at work (H. G. Gadamer, 2006).

### Strengths and limitations

A key strength of this study is that interviews with students took place at the end of the first year of university, allowing participants to reflect back over the course of the entire academic year and provide rich in-depth accounts of their experiences of flourishing. If this study had involved students speaking about their experiences at the beginning of first year, it is unlikely they would have spoken about the opportunities or resources on campus that promoted their flourishing, as most participants became more involved in University activities in the second semester.

There has been an increase in student diversity on Canadian campuses since the start of the 21^st^ century (Michalski et al., 2017) and although there was some diversity among the study participants there was an absence of international students transitioning to first year directly from high school in their country of origin. Students identifying with gender and or/sex identities other than male and female did not volunteer to participate in this study which is also a limitation of the study. Further, only one male student volunteered to take part in the study. Men typically participate less in qualitative health research (Affleck et al., 2013), and this may help to explain why more men did not volunteer for our study. The recruitment for this study also yielded many Bachelor of Nursing students, and there was limited diversity of participants in terms of programs of study. In spite of these limitations, however, our study does add to the knowledge base.

### Implications for practice, policy and research

Exploring the experiences of flourishing has provided insight into programs and policies that might be implemented in high schools and universities to ease this transition for those students who are struggling, and promote continued flourishing among those who are thriving. High schools could, for example, have mentoring programs whereby university students who have made the transition to university speak to high school students about their experiences with the transition. To facilitate this type of programming in rural and remote communities, videoconferencing could be arranged.

University life is not only concerned with academic development but achievement of developmental growth in the transitions is also central to the experience of flourishing at university. Fostering students’ personal strengths and capabilities (Davidson et al., 2009; Galassi, 2017; Xie, 2013), normalizing challenges experienced in first year, and encouraging students to take on challenges is critical to supporting academic success and wellbeing (Yeager & Dweck, 2012). Exploring ways of changing perceptions at all levels, from the individual student level to administrative levels at high school and university about the importance of “challenges” has the most promise for helping with the transition and encouraging students to flourish. It is also important that universities foster safe environments in which students feel comfortable asking for help to ease their transition. Asking for help was particularly challenging for many participants in this study and, ideally, learning this skill in the pre-transition period would better equip students entering their first-year university (Fainstad et al., 2018).

Opportunities within the university context for health promotion initiatives involving peer support might also contribute to flourishing in first year of university. Studies have found peer learning in undergraduate nursing programs to be effective in developing communication and critical thinking skills as well as boosting students’ self-confidence, with an added benefit of being a cost-effective way of having students support one another’s learning (Stone et al., 2013). Peer-led support for first-year students might involve students in their fourth year, sharing with students in first year the challenges they faced and how challenges were overcome, or by assigning first-year students a peer mentor who has transitioned to university and who can assist them in navigating the new environment (Miller et al., 2019).

Universities are invested in helping prepare students for the workforce which includes learning how to work with others and develop the necessary social and emotional skills to transition into the workforce. Although sometimes challenging for students, group work fosters social and emotional development (Laal & Ghodsi, 2012) and is key to academics and success in life (Kosterman et al., 2019). Learning these social and emotional skills in elementary and secondary schools might be beneficial in fostering social and emotional competence entering first-year university (Wyatt & Bloemker, 2013).

Faculty can also support students by having an awareness of common challenges faced by first-year students and understanding these challenges. Participants in our study were more likely to reach out to faculty who embodied qualities of openness, patience, acceptance and genuinely cared about their wellbeing. Faculty awareness of on-campus supports and resources is important but there is also a need for faculty to provide opportunities for students to feel safe discussing concerns, and supporting students to solve their own challenges.

In our study the midsized university and small class sizes provided opportunities to have meaningful relationships with faculty and peers which, as others have noted, promotes students’ sense of belonging to the university environment and their program of choice (Chapman & Ludlow, 2010) but responsibilities for easing the transition do not lie solely with universities and include families. Among some of the participants in our study, family and parental support was a significant resource for flourishing, yet family challenges were also present. High schools, and other stakeholder organizations such as community health centres, could provide education and support for parents who need it prior to their children making the transition to post-secondary education in order to help promote family and parental support. Parenting workshops aimed at fostering children’s strengths and educating parents on how to apply a strength-based parenting approach to deal with everyday challenges and managing the transition to university could feature in community programs.

Further qualitative studies involving other post-secondary contexts, such as at colleges and technology institutes might mean the discovery of additional factors associated with flourishing specific to these contexts and populations of students. Future studies could also target specific populations making critical transitions such as mature students making a career transition, or mature students with no previous post-secondary educational experience. Longitudinal studies are also of importance as they could explore the experiences of flourishing over time beginning in high school, throughout university, and then following students into the workforce. Longitudinal studies would help build an understanding of the experiences of flourishing over time and within changing contexts. Studies of this nature are adept at identifying pathways, meaning, turning points, changes and interpretive stances over the course of one's life,  (Hermanowicz, 2016).

## Conclusion

Our research explored the experience of flourishing among full-time students between the ages of 18–20 transitioning directly from high school to university. This transition may be challenging, however there are students who are able to flourish. Exploring the experiences of flourishing using Gadamer’s hermeneutics unearthed the individual, contextual, temporal and dialectical nature of flourishing during this transition. Further qualitative research is critical in order to advance our understanding of flourishing within other post-secondary contexts in light of the growing concern for student mental health.
